# Methodological Study on Determination of Recombinant Adeno-Associated Virus Particle Titer Through Size Exclusion Chromatography with Multiangle Light Scattering and Collaborative Calibration of Standard Substances

**DOI:** 10.3390/molecules30102170

**Published:** 2025-05-15

**Authors:** Dening Pei, Xiang Li, Hua Bi, Wenhong Fan, Heng Wang, Manli Cui, Xi Qin, Chenggang Liang

**Affiliations:** 1 National Institute for Food and Drug Control, No. 31 Huatuo St., Daxing District, Beijing 100050, China; 2 Waters Technology (Beijing) Co., Ltd., Building 1, No. 156, Jinghai 4th Road, Tongzhou District, Beijing 101111, China

**Keywords:** adeno-associated virus, viral particle titer, multiangle light scattering, size exclusion chromatography

## Abstract

Adeno-associated virus (AAV) is a promising gene therapy vector due to its high transduction efficiency, low pathogenicity, low immunogenicity, and the ability to mediate the long-term stable expression of exogenous genes. The viral particle titer is an essential quality attribute of recombinant adeno-associated virus (rAAV) gene therapy products. Multiangle light scattering (MALS) is an important means of directly measuring the absolute molecular weight and distribution of macromolecular drugs. This study established and validated a method based on SEC-UV-MALS-RI tandem technology for accurately determining rAAV particle titers. The verification results indicated that the method exhibited good specificity, linearity, precision, accuracy, and durability. Several collaborative laboratories used this method to calibrate the standard substances needed for rAAV particle titer determination. The results suggested that combining the SEC-MALS method with standard substances enables the rapid and accurate measurement of the viral particle titers in rAAV gene therapy products.

## 1. Introduction

Adeno-associated virus (AAV), a non-enveloped single-stranded DNA virus belonging to the Parvoviridae family, is characterized by an icosahedral protein capsid, a single-stranded DNA genome of 4.7 kb, and a diameter of 20~26 nm. There are 13 natural serum subtypes (AAV1-13) and nearly 200 variants of AAV. Recombinant adeno-associated virus vector (rAAV) is the most widely used viral vector in gene therapy due to its long-term gene expression in vivo, high tissue specificity, low immunogenicity, and safety [1,2]. The commonly used rAAV vectors in clinical applications include AAV2, AAV5, AAV8, and AAV9 serotypes. Currently, eight rAAV gene therapy products have been commercially approved worldwide, with over 200 clinical trials underway. These trials cover indications such as ophthalmic, neurological, metabolic, hematological, neuromuscular, and cardiovascular diseases, as well as tumors, demonstrating the potential of rAAV in treating various genetic and acquired diseases [3,4,5].

The viral particle titer (capsid titer) is a key quality attribute for rAAV gene therapy products. The dosage accuracy and the safety and effectiveness of drugs can only be guaranteed by obtaining an accurate and reliable viral particle titer [6]. At present, the commonly used method for determining viral particle titers is enzyme-linked immunosorbent assay (ELISA), which involves complex procedures and a long experimental period. Additionally, the standard samples used in ELISA tend to yield inaccurate and inconsistent results. To address these issues, it is necessary to develop an accurate and rapid method for determining viral particle titers and establish a standard substance for rAAV particle titer determination.

Multiangle light scattering (MALS) is an important technology for directly measuring the absolute molecular weight and distribution of large-molecule drugs, offering high accuracy and a wide measurement range. MALS has been widely applied in drug quality research [7,8]. When combined with size exclusion chromatography (SEC), ultraviolet (UV) detectors, and differential refractive index (RI) detectors, MALS can determine the absolute molecular weight and distribution of biomolecules while also characterizing their conformation and aggregation state. This technology provides accurate measurements of the molecular weight and empty shell rate of rAAV and has been used to determine the rAAV particle titer [9,10].

This study established and validated a method for accurately determining the rAAV particle titer using SEC-UV-MALS-RI tandem analysis. The method was used to calibrate a batch of standard substances for rAAV particle titer determination.

## 2. Results

### 2.1. Methodology Validation

#### 2.1.1. Specificity

**Figure 1:**
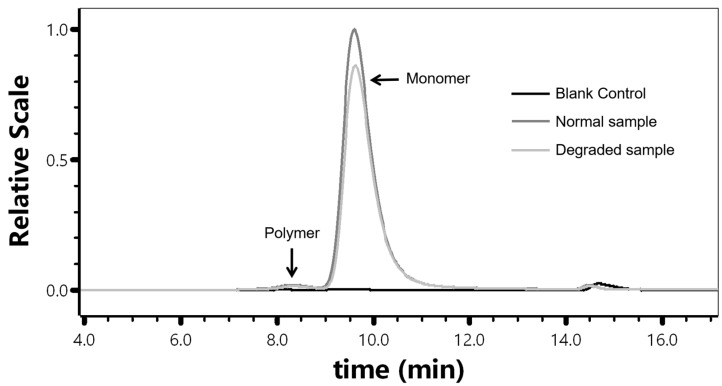
Chromatogram of blank control, normal sample, and degraded sample.

The blank control had no obvious interference peaks within the integration interval, and the resolution between the polymer and monomer peaks of the 100% concentration sample was ≥0.5. There was a significant difference between the spectra of degraded and normal samples (Figure 1).

#### 2.1.2. Repeatability of Multiple Injections

**Figure 2:**
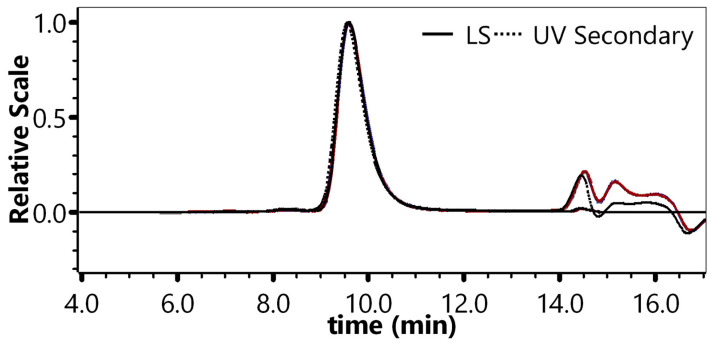
Chromatogram of six injections.

The monomer peak retention time for 6 injections ranged from 9.55 min to 9.60 min (RSD: 0.16%). The average monomer peak percentage was 97.53% (RSD: 0.20%) with a 95% confidence interval of 97.33–97.44%. The average monomer particle titer was 3.09 × 10^12^ VP·mL^−1^ (RSD: 2.97%) with a 95% confidence interval of 2.99 × 10^12^ VP·mL^−1^–3.18 × 10^12^ VP·mL^−1^. Thus, the results demonstrated high repeatability across multiple injections (Figure 2, Table S1).

#### 2.1.3. Repeatability of Multiple Preparations

**Figure 3:**
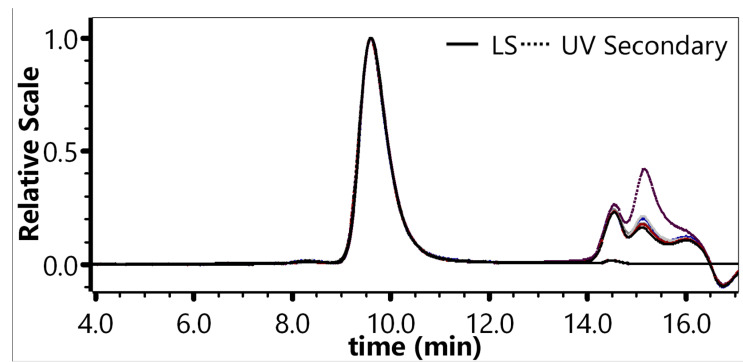
Chromatogram of six sample preparations.

The monomer peak retention for 6 sample preparations ranged from 9.58 min to 9.59 min (RSD: 0.023%). The average monomer peak percentage was 97.89% (RSD: 0.09%) with a 95% confidence interval of 97.80–97.98%. The average monomer particle titer was 2.87 × 10^12^ VP·mL^−1^ (RSD: 2.78%) with a 95% confidence interval of 2.78 × 10^12^ VP·mL^−1^–2.95 × 10^12^ VP·mL^−1^. These results demonstrated excellent preparation repeatability (Figure 3, Table S2).

#### 2.1.4. Intermediate Precision

**Figure 4:**
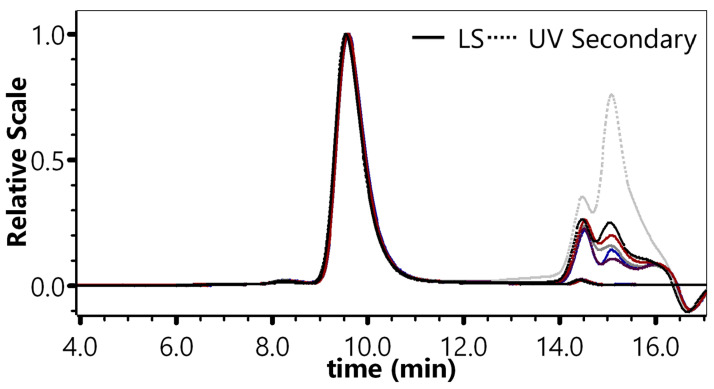
Chromatogram of six experiments, performed on different dates.

The monomer peak retention for 6 experiments conducted on different days ranged from 9.54 min to 9.59 min (RSD: 0.20%). The average monomer peak percentage was 97.76% (RSD: 0.11%) with a 95% confidence interval of 97.64–97.87%. The average monomer particle titer was 2.93 × 10^12^ VP·mL^−1^ (RSD: 3.40%) with a 95% confidence interval of 2.83 × 10^12^ VP·mL^−1^–3.04 × 10^12^ VP·mL^−1^. These results demonstrated excellent intermediate precision (Figure 4, Table S3).

#### 2.1.5. Accuracy

The recovery rates of five samples at different concentrations ranged from 99.99% to 112.39% (Table 1). These results demonstrated excellent accurary.

#### 2.1.6. LOD and LOQ

**Figure 5:**
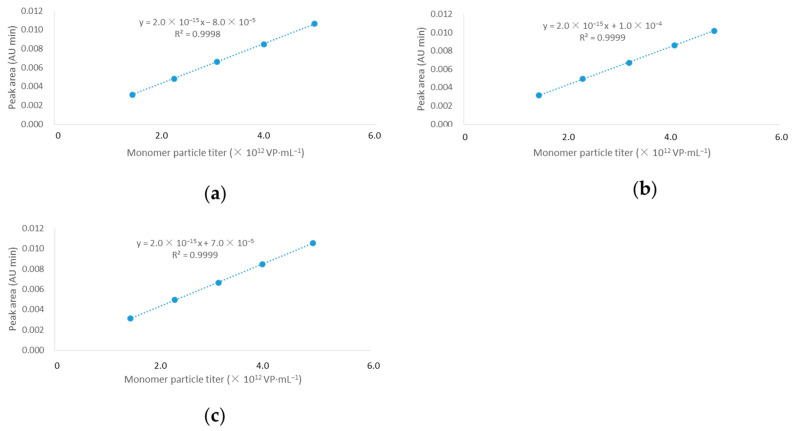
Regression lines between monomer particle titers and peak areas at different concentrations for run 1 (**a**), run 2 (**b**), and run 3 (**c**).

Samples for LOD and LOQ determination were prepared, loaded, and analyzed. The results showed that the LOD was 2.52 × 10^10^ VP·mL^−1^, and the LOQ was 7.64 × 10^10^ VP·mL^−1^ (Figure 5).

#### 2.1.7. Linearity and Range

The measurement values of particle titers met the requirements of accuracy, repeatability, and linearity within the concentration range of 50% to 150% of 2.87 × 10^12^ VP·mL^−1^ (Figure 5). Thus, the working range of this method was from 1.43 × 10^12^ VP·mL^−1^ to 4.30 × 10^12^ VP·mL^−1^.

#### 2.1.8. Sample Stability Analysis

The rAAV2 empty shell sample (150% concentration) was kept at 4 °C for 48 h. The results showed that |ΔT| was 0.033 min and D1% was 0.65%, indicating that the sample remained stable under these conditions (Table 2).

### 2.2. Collaborative Calibration Results

**Figure 6:**
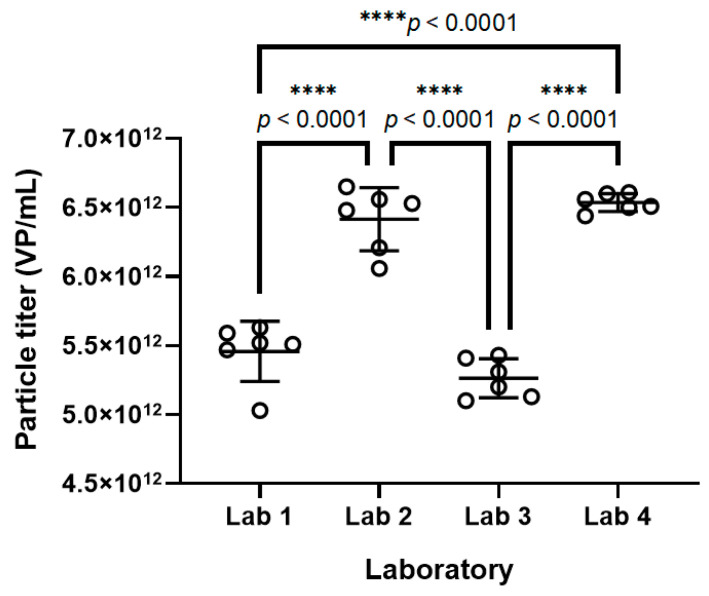
Collaborative calibration results of rAAV particle titers.

ANOVA demonstrated significant differences across laboratories; all pairwise comparisons except for Lab1–Lab3 and Lab2–Lab4 were statistically significant (*p* < 0.0001). Non-significant markers were excluded from the figure (Figure 6, Table S4). Across the four laboratories, the RSD was 10.40%, with an average of 5.84 × 10^12^ VP·mL^−1^ from 24 measurements. The 95% confidence interval was 5.61 × 10^12^ VP·mL^−1^–6.07 × 10^12^ VP·mL^−1^, and the 95% reference range was 4.74 × 10^12^ VP·mL^−1^–6.94 × 10^12^ VP·mL^−1^. Thus, 5.84 × 10^12^ VP·mL^−1^ was considered the reference value for standard substances in rAAV particle titer determination.

## 3. Discussion

Viral particle titers are important quality control indicators for gene therapy products involving viral vectors. Viral particle titers are analyzed throughout the development and clinical use of gene therapy products, directly affecting the safety and effectiveness of the products. Therefore, particle titer analysis not only provides the scientific basis for optimizing production processes and clinical doses but also serves as the foundation for regulatory compliance and promotes the standardization of gene therapy products.

Based on the multiangle light scattering principle, MALS technology calculates the molecular weight and size distribution of polymer particles by measuring the scattered light intensity at different angles. The aim is to use the scattering data at different angles to eliminate the influence of particle shape and size on the scattered signal, ensuring more accurate molecular weight information. This study utilized SEC-UV-MALS-RI technology, and the SEC-HPLC separated different sample components by size. The MALS detector provided the molecular weight data, enabling the differentiation of intact rAAV, empty shell rAAV, aggregates and degradation products. RI and UV detector signals were used to further determine the rAAV particle titer [11,12]. Before entering the light-scattering detector, samples are separated by SEC, which separates AAV monomers and polymers from some potential protein fragments and impurities. When analyzing samples, only the integral analysis of AAV monomer and polymer peaks can avoid the interference of protein fragments and impurities. For different types of AAV, the detection method is a general detection method, which can be easily applied to AAV8, AAV9, and other serotypes of AAV. When the AAV of different serotypes is determined by sec-uv-mals-ri, only different parameters need to be inputted, including the UV extinction coefficient of capsid and nucleic acid at 280 nm and 260 nm, respectively. The UV extinction coefficients of the capsid and nucleic acid of different serotypes of AAV will have some subtle differences, but the accurate parameters can be obtained by analyzing and calculating the UV and differential signals of AAV with Astra software (version 8.2.2.119). The difference in dn/dc values of capsid and nucleic acid of different serotypes of AAV is negligible relative to the systematic error, so it is usually a fixed value.

In method development, selecting a suitable chromatographic column is crucial since it determines whether the resolution of the SEC chromatographic column is sufficient to distinguish particles of similar sizes (such as empty shell and intact rAAV) and whether the chromatographic column has an adsorption effect on rAAV particles. The chromatographic column used in this study met these requirements. Additionally, the ionic strength of the mobile phase and the sample purity can affect the separation efficiency of SEC and should be carefully controlled. Moreover, careful attention should be paid to system correction and calibration due to their significance in accurate detection. In this study, the system was first balanced using a mobile phase. Then, the BSA standard solution was used for delay volume correction, peak broadening correction, the normalization constant correction of light scattering instrument, and the calibration of the UV detector constant. Additionally, system parameters must be calibrated, including the dn/dc value and extinction coefficients at 260 nm and 280 nm of rAAV capsid protein and nucleic acid DNA.

After development, the method was validated as per the requirements of ICH Q2. The method met the required criteria for specificity, precision, and accuracy. The LOD and LOQ of the method were 2.52 × 10^10^ VP·mL^−1^ and 7.64 × 10^10^ VP·mL^−1^, respectively, with a working range of 1.43 × 10^12^~4.30 × 10^12^ VP·mL^−1^. The sample showed good stability after 48 h at 4 °C, indicating good durability.

When SEC-MALS detects the number of AAV particles, the nucleic acids inside AAV absorb light; AAV generally comprises empty, partial, and full capsids. Different nucleic acids produce different light absorption values, affecting the accuracy of counting AAV particles. Here, we produced a batch of empty capsid standard substances, which were tested by AUC tests in multiple labs. The empty rate of this standard substance was 95.0 ± 2.6% [13]. The SEC-MALS method was used to calibrate the empty capsid standard, yielding the value of 5.84 × 10^12^ VP·mL^−1^. This empty capsid standard substance can be used as standard substance for rAAV particle titer determination. Using this standard substance for particle detection by SEC-MALS can improve the accuracy of the detection results. In future, this standard substance can be used as homogeneous standard substance for particles detection methods such as ELISA (using universal antibodies), as well as for the system suitability validation of SEC-MALS. We collected detection data from four labs, with a maximum intra-lab RSD of 4.0% and an inter-lab RSD of 10.4%, which met the requirement of ICH-Q2 (R2) for an RSD below 30% and verified the reproducibility of the method between laboratories.

SEC-MALS completes rAAV capsid titer analysis within 20 min, significantly faster than ELISA (>2 h, excluding antibody coating time). This rapid detection capability makes SEC-MALS more suitable for high-throughput sample analysis. EC-MALS quantifies capsid proteins based on their absolute molecular weight (~3.8 × 10^6^ Da) and hydrodynamic radius, independent of capsid surface epitopes, thereby eliminating serotype selectivity [9]. In contrast, ELISA relies on serotype-specific antibody-binding efficiency. Commercial ELISA kits are designed for wild-type capsids. For engineered serotypes, new antibodies must be screened and validated, substantially increasing both temporal and economic costs. Additionally, since the AAV capsid contains multiple antibody-binding epitopes (composed of three capsid subunits repeated in a specific stoichiometric ratio), ELISA quantification requires homologous reference standards calibrated by low-throughput methods like SDS-PAGE silver staining, whereas SEC-MALS enables direct quantification without reference standards. SEC-MALS integrates separation and detection modules within a fully automated chromatographic system, requiring only sample pretreatment and mobile phase preparation, which significantly reduces the risk of human-induced errors. In contrast, ELISA involves multi-step operations requiring stringent temperature and time controls, including incubation, repeated plate washing, and microplate reader parameter calibration. These intricate procedures heighten operator dependency and elevate inter-batch variability risks due to deviations in incubation timing or incomplete plate washing [14]. Although ELISA exhibits higher theoretical sensitivity (<1 × 10^9^ VP·mL^−1^) than SEC-MALS (~1 × 10^10^ VP·mL^−1^), its upper linear range (≤1 × 10^10^ VP·mL^−1^) is incompatible with typical rAAV production titers (often >1 × 10^12^ VP·mL^−1^), necessitating 10^2^–10^3^-fold dilutions. Such extensive dilution risks introducing matrix effects or pipetting errors. SEC-MALS, however, directly analyzes undiluted or minimally diluted samples (≤10-fold dilution), circumventing dilution-related inaccuracies while maintaining sufficient sensitivity for process monitoring.

In summary, SEC-MALS technology offers significant advantages for rAAV particle titer determination. As a multi-parameter analysis tool, it holds strong potential for the quality assessment of gene therapy products. Combining SEC-MALS with standard substances enables rapid and accurate rAAV particle titer determination in gene therapy products, making it a promising universal method for rAAV gene therapy products.

## 4. Materials and Methods

### 4.1. Reagents

Sodium dihydrogen phosphate, disodium hydrogen phosphate, and sodium chloride (purity ≥ 99.0%) were purchased from Sinopharm Group Chemical Reagent Co., Ltd. (Beijing, China). Bovine serum albumin (BSA, batch number A1900-250MG) was obtained from Sigma-Aldrich (Shanghai, China). A recombinant adeno-associated virus serotype 2 (rAAV2) empty shell sample (150% concentration) was prepared by the National Institutes for Food and Drug Control (Beijing, China).

### 4.2. Instrument

The experiment was conducted using Waters Arc high-performance liquid chromatography coupled with a PDA detector (Waters Corporation, Milford, MA, USA), a DAWN 18 angle static light scattering instrument, an Optilab interference differential refractometer, gel, and a chromatographic column (WTC-050N5, Wyatt Technology, Goleta, CA, USA, 4.6 mm × 300 mm, 5 μm 500 V or XBridge Premier GTx BEH SEC Column, Waters, Milford, MA, USA, 450 Å, 2.5 µm, 4.6 × 300 mm). Molecules were analyzed using ASTRA software (version 8.2.2.119) (Waters—WYATT Technology Corporation, Milford, MA, USA).

### 4.3. SEC-MALS Method

#### 4.3.1. Sample Preparation

The rAAV2 empty shell samples (4.30 × 10^12^ VP·mL^−1^, i.e., 150% concentration) were diluted with sample diluent to different concentrations (50%, 75%, 100%, and 125%) before use.

#### 4.3.2. Balance and Calibration of the System

The system balance was conducted using a 50 mmol·L^−1^ phosphate buffer (3.50 g/L sodium dihydrogen phosphate, 8.95 g/L sodium diphosphate dodecahydrate, and 20.44/L g sodium chloride; filter-sterilized using 0.22 μm filter) as the mobile phase. A 2 mg·mL^−1^ BSA solution (10 mg of BSA dissolved in 5 mL of the mobile phase) was used for system correction and calibration (referring to the instrument instructions with control limit ±5%), including detector delay correction, dilution effect correction, 18-angle light scatter normalization, and the calibration of UV detector constants.

#### 4.3.3. Chromatographic Conditions

The chromatographic conditions were set to a flow rate of 0.3 mL·min^−1^, a sample volume of 50 μL, a temperature of 30 °C, and a collection time of 35 min. The UV detector was set to 260 nm and 280 nm, while the 18-angle laser scattering and differential detection used default settings. Each sample was analyzed in duplicate. ASTRA software was used to analyze the UV signal, the 18-angle light scattering signal, and the interferometric differential refractometer signal data in order to calculate the particle titer.

### 4.4. SEC-MALS Methodology Validation

#### 4.4.1. Specificity

The blank control (sample diluent), the sample (100% concentration), and the degraded sample (100% concentration) damaged by repeated freeze–thaw cycles were analyzed simultaneously using the chromatographic conditions in Section 4.3. The corresponding chromatograms were recorded to investigate the specificity of the method.

#### 4.4.2. Repeatability of Multiple Injections

One sample (100% concentration) was injected 6 consecutive times under the chromatographic conditions in Section 4.3. The retention times and percentage of the monomer peak (main peak) were recorded, and the monomer particle titers were calculated to evaluate the injection repeatability. The results were considered acceptable if the RSD% for peak retention time was ≤5.0% and the RSD% for monomer particle titers was ≤5.0%.

#### 4.4.3. Repeatability of Multiple Preparations

Six samples (100% concentration) were prepared in parallel and analyzed under the chromatographic conditions in Section 4.3. The retention times and percentage of the monomer peak (main peak) were recorded, and the monomer particle titers were calculated to evaluate the preparation repeatability. The results were considered acceptable if the RSD% for peak retention time was ≤5.0% and the RSD% for monomer particle titers was ≤5.0%.

#### 4.4.4. Intermediate Precision

Six samples (100% concentration) were prepared and analyzed on different days under the chromatographic conditions in Section 4.3. The retention times and percentage of the monomer peak (main peak) were recorded, and the monomer particle titers were calculated to evaluate the preparation repeatability. The results were considered acceptable if the RSD% for peak retention time was ≤5.0% and the RSD% for monomer particle titers was ≤5.0%.

#### 4.4.5. Accuracy

Samples with different concentrations (50%, 75%, 100%, 125%, and 150%) were prepared, with three replicates per concentration. The samples were simultaneously analyzed under the chromatographic conditions in Section 4.3. The average values of monomer particle titers for the three replicates of each concentration were calculated and used as the measured value. The measured values of monomer particle titers of three replicates for the 100% concentration were taken as the expected values of 100% concentration samples. The expected values of particle titer of the other sample concentrations were calculated according to the dilution ratio. The accuracy of each concentration sample was calculated based on the recovery rate: recovery rate (%) = (the measured values of particle titer/the expected value of particle titer) × 100%.

The method was considered acceptable if recovery rates for all five concentrations were within 80–120%.

#### 4.4.6. Limit of Detection (LOD) and Limit of Quantitation (LOQ)

Three samples with different concentrations (50%, 75%, 100%, 125%, and 150%) were prepared, as described in Section 4.4.5. The monomer particle titer and the corresponding monomer peak area were obtained after sample loading and analysis. Three linear regression equations were obtained: y = kx + b (where y is the monomer peak area and x is the monomer particle titer). The standard deviation (δ) of the intercept b and the average (S) of the slope k of the three equations were calculated. The LOD and LOQ were calculated using the standard deviation of the response and the standard curve slope methods: LOD = 3.3δ/S; LOQ = 10δ/S.

#### 4.4.7. Linearity and Range

As described in Section 4.4.5, linear regression analysis was conducted between the monomer particle titers of samples with different concentrations (50%, 75%, 100%, 125%, and 150%) and their corresponding peak areas, yielding R^2^ > 0.999. Meanwhile, the measured values of monomer particle titers at all concentrations (50%, 75%, 100%, 125%, and 150%) met the accuracy and repeatability requirements. Therefore, the working range of this method is 50–150%.

#### 4.4.8. Sample Stability Analysis

The rAAV2 empty shell samples (150% concentration) were kept at 4 °C and analyzed at 0 h, 24 h, and 48 h time points under the chromatographic conditions in Section 4.3. The monomer peak retention time and monomer particle titer were recorded. The difference in monomer peak retention time (ΔT) and monomer particle titer (D%) were calculated as follows:

ΔT = |A − B|, where A is the monomer peak retention time at 0 h, and B is the monomer peak retention time at 24 h or 48 h.

D% = (|D − C|/C) × 100%, where C is the monomer particle titer at 0 h, and D is monomer particle titers at 24 h or 48 h.

Acceptable criteria: ΔT ≤ 0.5; D% ≤ 10.0%.

### 4.5. Collaborative Calibration

Four collaborating laboratories were organized, each performing six measurements of rAAV empty shell samples at 150% concentration using the chromatographic conditions described in Section 4.3. The inter-laboratory precision (reproducibility) of the method was investigated. The average value of 24 monomer particle titer measurements was used as the reference value for the standard substances in rAAV particle titer determination.

## Figures and Tables

**Table 1:** Recovery rates of five samples at different concentrations.

	Monomer Particle Titer (VP·mL^−1^)	Peak Area (AU min)	The Expected Value of Monomer Particle Titer (VP·mL^−1^)	Recovery Rate (%)
50% concentration sample—run 1	1.45 × 10^12^	3.13 × 10^−3^	1.43 × 10^12^	
50% concentration sample—run 2	1.42 × 10^12^	3.13 × 10^−3^	1.43 × 10^12^	
50% concentration sample—run 3	1.43 × 10^12^	3.12 × 10^−3^	1.43 × 10^12^	
Average value	1.43 × 10^12^	3.13 × 10^−3^	1.43 × 10^12^	99.99
RSD (%)	1.07	0.18		
75% concentration sample—run 1	2.23 × 10^12^	4.82 × 10^−3^	2.15 × 10^12^	
75% concentration sample—run 2	2.26 × 10^12^	4.92 × 10^−3^	2.15 × 10^12^	
75% concentration sample—run 3	2.28 × 10^12^	4.94 × 10^−3^	2.15 × 10^12^	
Average value	2.26 × 10^12^	4.89 × 10^−3^	2.15 × 10^12^	104.94
RSD (%)	1.12	1.31		
100% concentration sample—run 1	3.02 × 10^12^	6.58 × 10^−3^	2.87 × 10^12^	
100% concentration sample—run 2	3.13 × 10^12^	6.69 × 10^−3^	2.87 × 10^12^	
100% concentration sample—run 3	3.11 × 10^12^	6.65 × 10^−3^	2.87 × 10^12^	
Average value	3.09 × 10^12^	6.64 × 10^−3^	2.87 × 10^12^	107.70
RSD (%)	1.90	0.84		
125% concentration sample—run 1	3.90 × 10^12^	8.47 × 10^−3^	3.58 × 10^12^	
125% concentration sample—run 2	4.00 × 10^12^	8.61 × 10^−3^	3.58 × 10^12^	
125% concentration sample—run 3	3.95 × 10^12^	8.46 × 10^−3^	3.58 × 10^12^	
Average value	3.95 × 10^12^	8.51 × 10^−3^	3.58 × 10^12^	110.25
RSD (%)	1.27	0.99		
150% concentration sample—run 1	4.84 × 10^12^	1.064 × 10^−2^	4.30 × 10^12^	
150% concentration sample—run 2	4.75 × 10^12^	1.017 × 10^−2^	4.30 × 10^12^	
150% concentration sample—run 3	4.90 × 10^12^	1.053 × 10^−2^	4.30 × 10^12^	
Average value	4.83 × 10^12^	1.044 × 10^−2^	4.30 × 10^12^	112.39
RSD (%)	1.56	2.35		

**Table 2:** Analysis results of the sample after being placed at 4 °C.

	Monomer Particle Titer (VP·mL^−1^)	Retention Time of Monomer Peak (min)
0 h	2.71 × 10^12^	9.60
24 h	2.79 × 10^12^	9.59
48 h	2.72 × 10^12^	9.56

## Data Availability

The original contributions presented in this study are included in the article/Supplementary Materials. Further inquiries can be directed to the corresponding authors.

## Supplementary Materials

The following supporting information can be downloaded at: https://www.mdpi.com/article/10.3390/molecules30102170/s1, Table S1: Analysis scripts of repeatability of multiple injections; Table S2: Analysis scripts of repeatability of multiple preparations; Table S3: Analysis scripts of intermediate precision; Table S4: Analysis scripts of collaborative calibration results.

## Abbreviations

The following abbreviations are used in this manuscript:

AAV	adeno-associated virus
SEC-MALS	size exclusion chromatography with multiangle light scattering
RI	refractive index
ELISA	enzyme-linked immunosorbent assay
RSD	relative standard deviation
LOD	limit of detection
LOQ	limit of quantitation
