# Supplementary material for: Methodological Study on Determination of Recombinant Adeno-Associated Virus Particle Titer Through Size Exclusion Chromatography with Multiangle Light Scattering and Collaborative Calibration of Standard Substances
Source: Molecules. 2025 May 15;30(10):2170. doi: 10.3390/molecules30102170 (PMC12114095; doi:10.3390/molecules30102170)
Supplement: Supplementary file 1 [file molecules-30-02170-s001.zip › molecules-3588218-supplementary.pdf]

Table S1. Analysis scripts of repeatability of multiple injections

	Monomer peak retention time (min)	Aggregate peak retention time (min)	Percentage of monomer peak area (%)	Monomer particle titer (VP·mL⁻¹)	Aggregate particle titer (VP·mL⁻¹)	Total particle titer (VP·mL⁻¹)
Injection-1	9.595	8.274	97.535	3.0396e+12	3.6554e+10	3.0762e+12
Injection-2	9.592	8.259	97.620	3.0799e+12	4.8928e+10	3.1288e+12
Injection-3	9.591	8.336	97.402	3.0697e+12	3.9211e+10	3.1089e+12
Injection-4	9.590	8.353	97.719	3.0310e+12	3.3043e+10	3.0640e+12
Injection-5	9.589	8.316	97.213	3.0371e+12	5.8829e+10	3.0959e+12
Injection-6	9.554	8.259	97.717	3.2707e+12	3.7193e+10	3.3079e+12

Table S2. Analysis scripts of repeatability of multiple preparations

	Monomer peak retention time (min)	Aggregate peak retention time (min)	Percentage of monomer peak area (%)	Monomer particle titer (VP·mL⁻¹)	Aggregate particle titer (VP·mL⁻¹)	Total particle titer (VP·mL⁻¹)
Preparation -1	9.587	8.314	97.898	2.7480e+12	2.0537e+10	2.7685e+12
Preparation -2	9.588	8.357	97.940	2.8696e+12	1.5467e+10	2.8851e+12
Preparation -3	9.585	8.337	97.962	2.8928e+12	1.5631e+10	2.9084e+12
Preparation -4	9.591	8.314	97.866	2.9876e+12	1.9523e+10	2.9938e+12
Preparation -5	9.586	8.268	97.951	2.8190e+12	1.5579e+10	2.8346e+12
Preparation -6	9.589	8.327	97.736	2.8778e+12	2.8895e+10	2.9067e+12

Table S3. Analysis scripts of intermediate precision

	Monomer peak retention time (min)	Aggregate peak retention time (min)	Percentage of monomer peak area (%)	Monomer particle titer (VP·mL^{-1})	Aggregate particle titer (VP·mL^{-1})	Total particle titer (VP·mL^{-1})
Day-1	9.586	8.299	97.910	3.0523e+12	2.2435e+10	3.0747e+12
Day -2	9.582	8.303	97.647	3.0423e+12	3.4904e+10	3.0772e+12
Day -3	9.573	8.253	97.704	2.9480e+12	2.9832e+10	2.9778e+12
Day -4	9.579	8.270	97.635	2.8468e+12	3.6587e+10	2.8833e+12
Day -5	9.548	8.272	97.809	2.8251e+12	2.7812e+10	2.8503e+12
Day -6	9.542	8.275	97.838	2.8680e+12	2.5663e+10	2.8937e+12

Table S4. Analysis scripts of collaborative calibration results

	Lab 1	Lab 2	Lab 3	Lab 4
Assay 1	5.03E+12	6.06E+12	5.10E+12	6.50E+12
Assay 2	5.47E+12	6.48E+12	5.13E+12	6.56E+12
Assay 3	5.51E+12	6.56E+12	5.41E+12	6.44E+12
Assay 4	5.63E+12	6.65E+12	5.43E+12	6.51E+12
Assay 5	5.52E+12	6.53E+12	5.20E+12	6.61E+12
Assay 6	5.59E+12	6.21E+12	5.31E+12	6.60E+12
